# Flower–bee versus pollen–bee metanetworks in fragmented landscapes

**DOI:** 10.1098/rspb.2023.2604

**Published:** 2024-05-29

**Authors:** Felipe Librán-Embid, Ingo Grass, Carine Emer, Viviana Alarcón-Segura, Hermann Behling, Siria Biagioni, Cristina Ganuza, Celina Herrera-Krings, Christina Ani Setyaningsih, Teja Tscharntke

**Affiliations:** ^1^ Agroecology, University of Göttingen, Göttingen 37077, Germany; ^2^ Justus Liebig University of Gießen, Institute of Animal Ecology and Systematics, Heinrich-Buff-Ring 26, Gießen 35390, Germany; ^3^ Department of Ecology of Tropical Agricultural Systems, University of Hohenheim, Stuttgart 70599, Germany; ^4^ Center for Biodiversity and Integrative Taxonomy (KomBioTa), University of Hohenheim, Stuttgart 70599, Germany; ^5^ Instituto de Pesquisas Jardim Botânico do Rio de Janeiro, Rua Pacheco Leão, 915, Jardim Botânico, Rio de Janeiro CEP22460-030, Brazil; ^6^ Animal Ecology, Department of Biology, University of Marburg, Marburg 35037, Germany; ^7^ Department of Palynology and Climate Dynamics, Albrecht‐von‐Haller‐Institute for Plant Sciences, University of Göttingen, Göttingen 37077, Germany; ^8^ Department of Animal Ecology and Tropical Biology, University of Würzburg, Würzburg 97074, Germany

**Keywords:** plant–pollinator interactions, calcareous grasslands, habitat fragmentation, pollen-transport networks, landscape ecology, life-history traits

## Abstract

Understanding the organization of mutualistic networks at multiple spatial scales is key to ensure biological conservation and functionality in human-modified ecosystems. Yet, how changing habitat and landscape features affect pollen–bee interaction networks is still poorly understood. Here, we analysed how bee–flower visitation and bee–pollen-transport interactions respond to habitat fragmentation at the local network and regional metanetwork scales, combining data from 29 fragments of calcareous grasslands, an endangered biodiversity hotspot in central Europe. We found that only 37% of the total unique pairwise species interactions occurred in both pollen-transport and flower visitation networks, whereas 28% and 35% were exclusive to pollen-transport and flower visitation networks, respectively. At local level, network specialization was higher in pollen-transport networks, and was negatively related to the diversity of land cover types in both network types. At metanetwork level, pollen transport data revealed that the proportion of single-fragment interactions increased with landscape diversity. Our results show that the specialization of calcareous grasslands’ plant–pollinator networks decreases with landscape diversity, but network specialization is underestimated when only based on flower visitation information. Pollen transport data, more than flower visitation, and multi-scale analyses of metanetworks are fundamental for understanding plant–pollinator interactions in human-dominated landscapes.

## 1. Introduction

Plant–pollinator interactions are the ecological foundations of animal-mediated pollination, a process on which 88% of flowering plants depend [1,2]. The structural analysis of pollination interaction networks may provide key information on network stability and robustness under environmental change [3–5]. Landscape-scale simplifications by habitat loss and habitat fragmentation have strong negative impacts on the characteristics of plant–pollinator network structure and functionality [3,6,7], directly through non-random loss of interactions [8] and indirectly through changes in species richness and abundance [9,10].

Land-use consequences on ecological networks must be explored at local and regional spatial scales because of the occurrence of important nonlinear emergent properties that demand the use of methods that can deal with multiple levels of ecological complexity [3,11,12]. Metanetworks (i.e. a group of scattered local networks connected by shared interactions) are an emerging approach to study the consequences of habitat fragmentation on ecological networks at a regional scale [10,12,13].

Plant–pollinator networks have been traditionally constructed using data on flower visitation [14]. However, in order for successful pollination to occur, viable pollen grains need to be transported from the anthers of a flowering plant to a receptive stigma of a conspecific [15]. Therefore, the sole flower visitation of an animal is expected to be a poor predictor of its capacity as a pollinator [16]. For instance, many flower visitors forage exclusively for nectar and do not contact flower anthers; other species lack morphological traits to carry pollen and thus cannot act as pollinators [17,18]. Two methods have been proposed to overcome this challenge. First, stigmas and styles can be analysed to identify pollen deposition after an animal visit [19,20]. However, pollen deposition analyses are extremely time-consuming and consequently prohibitive for landscape-scale studies. Alternatively, pollen loads analyses of flower visitors also provide valuable information regarding an animal’s capacity as a pollinator and are suitable for large-scale studies given their relative simplicity [21,22]. Pollen-transport networks have been studied at singular sites and local scales [23–26] and pollen metanetworks across land-use types [27], but, to our knowledge, there is no study analysing pollen-transport networks and metanetworks over gradients of habitat size, isolation and landscape diversity.

To compare the structure of different types of networks, such as flower visitation and pollen-transport interaction networks—between each other and across environmental gradients—it is essential to quantify network specialization at the community level [28]. This can be done through qualitative and quantitative indices such as network connectance and Blüthgen’s '*H*_2_' [28]. The specialization of pollination networks can be higher than that of visitation networks [23,29] given that the pollen richness on the bodies of flower visitors is usually a subset of the flowers they visit [30]. However, pollen analyses can also reveal interactions established with rare and unfrequently visited plant species that could lead to higher pollen-transport network connectance [22,31] and lower network specialization [32,33]. The specialization of mutualistic networks is affected by habitat loss and isolation through species turnover [34] and through a shift towards a higher prevalence of opportunistic interactions among generalists [35].

Species traits can determine the probability that a flower visit is accompanied by pollen transport and therefore that a certain interaction gets recorded with flower visitation observations, pollen load analyses or both. For example, given that bumblebees are social, usually hairier, larger and more numerous than other bees, they are generally expected to have a larger carrying capacity for pollen transport than most solitary bees in Europe [36]. Therefore, interactions established by bumblebees should have a higher probability of occurrence in both visitation and pollen-transport networks than those established by other bees. Furthermore, habitat specialist plants, rather than habitat generalists, often have adaptations to maximize visitation and amount of pollen transferred to pollinators per visit, such as larger floral displays (i.e. increased attractiveness [37]). Hence, habitat specialist plants should establish interactions with a higher probability of occurrence in both types of networks.

Mutualistic metanetworks in fragmented landscapes are characterized by a large majority of interactions that are unique to single fragments [11,12]. In a mutualistic metanetwork (*sensu* [12]), single-fragment interactions are those plant–bee interactions that are recorded in only one of the natural or seminatural studied habitat fragments. Based on flower visitation data of bees and butterflies in calcareous grasslands, Librán-Embid *et al*. [11] found that landscape diversity had a positive effect on the richness of single-fragment interactions and a negative effect on the proportion of single-fragment interactions. However, whether this effect is also captured with bee pollen loads analyses is still unknown.

Here, we tested how habitat fragmentation affects plant–pollinator interactions at four distinct but complementary levels of biological organization, i.e. (i) flower visitation and (ii) pollen-transport interactions at the (iii) local network and (iv) regional metanetwork scales.

We studied a gradient of habitat change of European calcareous grasslands, a highly threatened biodiversity hotspot characterized by a vast number of rare and endangered species [38]. Specifically, we asked (i) at the local scale, how landscape characteristics (fragment area, connectivity and landscape diversity of cover types) would shape the structure (connectance and specialization) of visitation and pollen-transport networks, and (ii) at the regional scale, which functional traits (i.e. bee body size, bee group, bee habitat specialization, flower size and plant habitat specialization) are associated with the probability that a flower visit involves pollen transport and how are single-fragment interactions affected by fragmentation and landscape diversity in both metanetworks.

We hypothesized that (i) the specialization of light-microscopy local pollen-transport networks will be higher than that of local visitation networks and their connectance lower. This is expected if the flower visitors’ morphological and behavioural constraints to pollen transport prevail over the emergence of interactions established with rare and unfrequently visited plant species; (ii) network specialization will decrease in larger and less isolated calcareous grasslands that are surrounded by more diverse land cover types, as the presence of more species should increase the probability of multiple interacting partners; (iii) owing to rare and ineffective interactions (i.e. interactions that contribute little to the plant reproductive success (*sensu* [15])), a high number of interactions unique to the pollen-transport and visitation metanetworks, respectively, is expected; (iv) interaction occurrence in both network types simultaneously depends on bee and flower size, bee and plant habitat specialization and bee group [11]; and finally, (v) the richness and proportion of single-fragment interactions increases with landscape diversity and fragment area and decreases with fragment connectivity in both types of networks given the central-place foraging behaviour of bees.

## 2. Methods

### (a) Study system

Calcareous grasslands in central Europe mainly result from low-intensity grazing activities associated with human livestock since the Anthropocene and are therefore considered seminatural habitats [38]. In the absence of extensive grazing, bush encroachment leads to the degradation of this formerly common and extended habitat [38]. In recent decades, agricultural intensification and land-use change have largely determined the reduction and fragmentation of this biodiverse habitat that harbours the highest richness of vascular plants, butterflies and grasshoppers in central Europe [39–42]. Due to these characteristics, calcareous grasslands are considered core habitats and conservation priorities in Europe and are therefore legally protected in the European Union [43,44].

### (b) Study area

Data were collected from April until August 2018 on 29 calcareous grasslands in the surroundings of the city of Göttingen, Germany (electronic supplementary material, figure S1). These grasslands were selected in a previous study [45], from a larger regional pool (~300 fragments), to vary along independent gradients of size and isolation from other calcareous grasslands. Arable land and European beech (*Fagus sylvatica*) forests are the two main land use types in the region, with 31% and 38% land cover, respectively [45].

### (c) Flower visitation interactions

We performed three rounds of sampling throughout the season in each calcareous grassland to capture the succession of flower visitors (hereafter, pollinators) and wildflower species. Seven fixed-point observation plots of 10 min were established at each site. We followed a protocol established by van Swaay *et al*. [46] to carry out our surveys. We collected data from 9.00 to 17.00 on days with a minimum temperature of 15°C and at least 50% clear sky, or with a minimum temperature of 18°C in any sky condition. To avoid any confounding effect of daytime, sites were surveyed at different times of the day.

Our observational plots were established in flower-rich areas and were circular (3 m radius, 28.3 m^2^). Within these, all interactions between bees (Hymenoptera: Apiformes) and flowering plants were recorded. We focused on bees because they are (jointly with lepidopterans) the most abundant pollinators in grasslands [47] and because they carry significantly larger pollen loads than butterflies [23]. A bee visit was considered an interaction once the insect contacted the plant’s reproductive organs. Bees that were not easily recognizable from a distance were sweep netted or collected for later identification by taxonomists and the timer was stopped while handling insects. We excluded interactions involving *Apis mellifera* because the presence of this species in our region relates exclusively to the occurrence of bee keepers in the vicinity [11]. *Apis mellifera* interactions accounted for 334 from a total of 1499 interactions registered and were present in all sites (range 1–75 *A*. *mellifera* interactions per site). Bees were classified as solitary bees or bumblebees (hereafter, bee group). All bumblebees are eusocial and belong to the genus *Bombus* spp. Within the group of ‘solitary bees,’ seven species presented some degree of sociality but were grouped within the solitary bees because of the morphological and genetic similarities with these. The seven species are *Andrena scotica* (communal), *Halictus confusus*, *Halictus rubicundus*, *Halictus tumulorum*, *Lasioglossum calceatum*, *Lasioglossum morio* and *Lasioglossum pauxillum*.

### (d) Pollen-transport interactions

Pollen was taken from bees’ bodies, head and antennae by bathing bees in Eppendorf tubes filled with distilled water, using a modified protocol from Dafni [48]. As some interactions were very abundant, we collected pollen from all bees that visited flowers in our observation plots, with an upper limit of collecting six pollen samples from the same interaction in each site and sampling round following Zhao et al. [25]. Pollen baskets were considered following de Manincor et al. [49], who found that there is no significant difference in the number of observed links between analyses based on pollen passively transported on the body and that collected in specialized structures such as the corbiculae. Samples were later acetolysed [50] using a protocol lab technique and analysed using light microscopy at 40× magnification (one slide represented one bee). We also created a reference collection of pollen from the flowering plants of the region to aid sample pollen identification. We did not consider slides with fewer than 30 pollen grains. From all the others, we counted 200 pollen grains on each slide, except for five slides that had 50–200 pollen grains. To ensure that pollen diversity was captured, the slides were scanned systematically in consecutive horizontal lines starting from the left upper corner of each slide and up to the count of 200 pollen grains. Following Bosch *et al*. [22], we considered the presence of at least 10 pollen grains in our samples as proof of true visitation to the corresponding flowering species (i.e. threshold for pollen–bee interactions).

### (e) Plant–pollinator traits

Plants and pollinators were classified according to their habitat specialization, following Poschlod *et al*. [51] and Brückmann *et al*. [52] for plants and Jauker *et al*. [53] and Hopfenmüller *et al*. [54] for bees. All body length values for bees were taken from Westrich [55]. We consider *Cirsium* sp. (cluster of four species mostly represented by the habitat specialist *Cirsium acaule*) and *Ononis* sp. (cluster of two hybridizing species including the specialist *Ononis repens*) as habitat specialists (i.e. species mostly restricted to available calcareous grassland habitat fragments).

### (f) Landscape metrics

We tested the effects of fragment size, fragment connectivity and landscape diversity of cover types on the structure of local fragment networks in terms of specialization (i.e. network connectance and H_2_') and also on the richness and proportion of single-fragment interactions (i.e. interactions that were only recorded in a single fragment). Fragment area was calculated with ArcGis 10.5.1 [56] and ranged from 82 to 52 557 m², excluding zones dominated by shrubs. Fragment spatial connectivity and the Shannon diversity of land cover types (as a measure of landscape diversity) were calculated using the ‘landscapemetrics’ package [57]. For fragments’ spatial connectivity, we used a connectivity index developed by Hanski *et al*. [58] and considered all calcareous grasslands in a radius of 2 km around the study grasslands (see electronic supplementary material for details). Larger values of this index indicate higher spatial connectivity (electronic supplementary material, table S1). The mapped cover types were: oilseed rape, grainfield, maize, other crops, forest open, forest closed, field margin, hedgerow, pasture, calcareous grassland, orchard, settlements, water bodies, streets, grass-roads and bare soil (see figure S1 in the electronic supplementary material). The basemap for habitat classification was provided by ‘the rural development and agricultural promotion service centre’ (Servicezentrum Landentwicklung und Agrarförderung). Shapefiles of land use were constructed using ArcGis 10.5.1 and all statistics were performed in R 4.1.0 [59].

### (g) Network and statistical analysis

Our study involved two levels of biological organization: (i) local scale, in which each of the 29 calcareous grasslands corresponds to a local network of both flower-visitation and pollen transport interactions, totalling 58 networks; and (ii) regional scale, in which we scaled-up from local fragment networks to regional metanetworks of both flower-visitation and pollen–transport interactions. Below, we describe how we analysed that complexity in the light of our hypotheses.

We constructed local quantitative bipartite networks (one for each calcareous grassland fragment) and regional metanetworks using data on flower visitation (hereafter, visitation networks) and pollen loads (hereafter, pollen-transport networks), respectively. Local networks were constructed as A*_ij_* adjacency matrices in which *i* are the plant species, *j* the pollinator species and the a*_ij_* element represents the frequency of interactions between *i* and *j*. At the landscape level, metanetworks were built by pooling the 29 calcareous grasslands into A*_kl_* adjacency matrices in which *k* are the studied sites and *l* either the plant visitation or the pollen-transport interactions. To make visitation and pollen-transport networks comparable we did not consider pollen from trees (e.g. *Picea* spp. or *Pinus* spp.), crops (e.g. *Vicia faba*), grasses (e.g. Poaceae) or ornamental plants (e.g. *Astrantia major*) because observations were done exclusively on herbaceous plants of calcareous grasslands.

### (h) Local networks

To test whether pollen-transport networks were more specialized than visitation networks at the local level (hypothesis 1) and whether they were affected by habitat fragmentation and landscape diversity (hypothesis 2), we calculated network connectance, defined as ‘the realized proportion of possible links’ and the H_2_' index, which is based on ‘the deviation of a species’ realized number of interactions expected from each species’ total number of interactions’ [28,60,61]. We used a linear mixed model with network type, (log) fragment area, (log) connectivity index and landscape diversity at 350 m as explanatory variables, and fragment identity as random intercept. To choose the spatial scale surrounding the focal calcareous grassland habitats at which landscape diversity effects were stronger, we compared models fitted at all scales from 100 to 500 m in 50 m intervals and compared them using the corrected Akaike information criterion (AICc) for small samples. Because almost all indices of network structure are at least partially affected by network size, we standardized our response variables, network connectance (more affected by network size) and H_2_' (less affected by network size) relative to a null model to allow for meaningful comparisons among networks of different fragments [61,62]. We followed Grass *et al*. [3] by creating null distributions based on 1000 replicates of Patefield’s algorithm. All network metrics were calculated using the ‘bipartite’ package [63] and model diagnostics was done using the ‘DHARMa’ package [64].

### (i) Metanetworks

We modelled the simultaneous presence of interactions in both metanetworks (hypothesis 4, i.e. flower visitation interactions resulting in pollen transport) using a generalized linear mixed model with binomial distribution and pollinator and plant species identity as crossed random intercepts. Our explanatory variables in the full additive model above were the plant and pollinator habitat specialization, flower size (area), pollinator size and group (i.e. bumblebee or solitary bee). Finally, to study the effects of landscape diversity and habitat fragmentation (i.e. fragment area and connectivity) on the richness and proportion of single-fragment interactions (hypothesis 5), we used generalized linear models with negative binomial distribution and linear models (i.e. normal distribution), respectively. The explanatory variables tested in the full models were the Shannon diversity index of land cover types at 150 m (for single-fragment interaction richness) and 500 m (for the proportion of single-fragment interactions), (log) fragment area and (log) connectivity index. The minimum adequate models were found with backwards model selection using likelihood ratio tests. All non-significant explanatory variables (*p* > 0.05) were sequentially removed. Models were created using the ‘lme4’ package [65]. All network and statistical analyses were performed in R 4.1.0 [59] .

## 3. Results

We observed 1165 flower–bee interaction events among 71 plant species and 67 bee species, resulting in 250 unique pairwise interactions. Of those, 31 (43.7%) plant species were only visited by bumblebees and 19 (26.8%) plant species were only visited by solitary bees, while 23 (32.4%) plant species were visited by both, totalling 71 plant species visited (electronic supplementary material, figure S2*a*, table S2). Some examples include *Fragaria vesca,* which was only visited by solitary bees, and *Trifolium pratense, Salvia pratensis, Prunella grandiflora, Carlina vulgaris* and *Anthyllis vulneraria,* which were only visited by bumblebees (electronic supplementary material, table S2). Furthermore, we analysed pollen samples of 830 bee individuals and found 474 individuals carrying 0–30 pollen grains, 5 carrying 50–200 pollen grains and 351 carrying ≥200 pollen grains. We identified 44 bee species transporting pollen from 64 plant species, resulting in 222 unique pollen–bee pairwise interactions from a total of 626 pollen interaction events. Pollen of 20 (31.3%) plant species was only transported by bumblebees and pollen of 12 (18.8%) plant species was exclusively transported by solitary bees (electronic supplementary material, figure S2*b*, table S3), while pollen of 32 (50%) plant species was transported by both groups. For example, pollen of *Knautia arvensis* was only transported by bumblebees and pollen of *Potentilla* sp. was only transported by solitary bees (electronic supplementary material, table S3).

**Figure 1.**
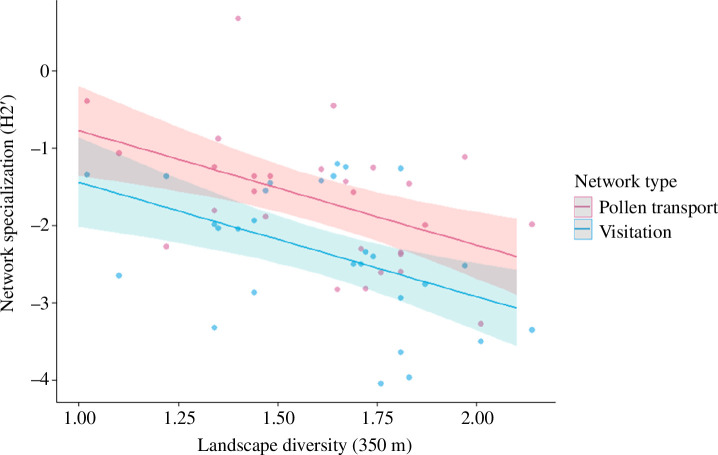
Relationship between standardized network specialization (*h_2_'*), network type and landscape diversity (i.e. Shannon diversity of land cover types). Each network type includes 28 local networks (fragments) in each dataset (pollen transport and visitation). Bands represent 95% confidence intervals of the model fit.

At the local network level, our results show that pollen-transport networks were significantly more specialized than visitation networks (*F*_1,27_ = 11.33, *p* = 0.002, figure 1). We also found a negative effect of landscape diversity at the 350 m scale on H_2_' specialization of both visitation and pollen-transport networks (*F*_1,26_ = 13.56, *p* = 0.001, figure 1). On the other hand, connectance did not differ between the visitation and pollen-transport networks (*F*_1,27_ = 1.03, *p* = 0.32) and was also not affected by landscape diversity (*F*_1,26_ = 1.97, *p* = 0.17). Fragment area and fragment connectivity had no significant effects neither on H_2_' network specialization nor connectance (electronic supplementary material, table S4).

**Figure 2.**
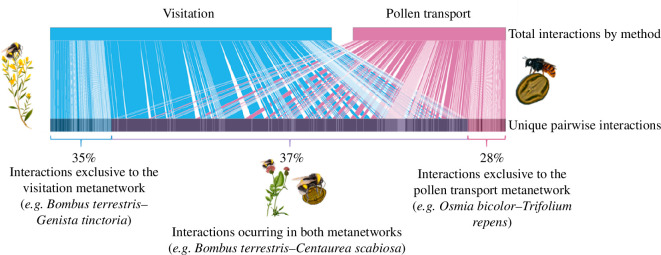
Bipartite diagram representation of the overlap between the visitation and pollen-transport metanetworks. The rectangles in the upper part represent the total interactions detected by each method (1165 and 626 total interactions in the visitation and pollen-transport metanetworks, respectively). The rectangles at the bottom represent the unique flower–bee (sky blue) and pollen–bee (pink) pairwise interactions. The size of the squares and thickness of the bars are proportional to the frequency of each unique pairwise interaction. Those interactions exclusive to the pollen transport dataset occur to the right (in pink) and those exclusive to the visitation dataset are shown to the left (in sky blue). Unique pairwise interactions occurring in both datasets are in the middle and highlighted in violet. The percentage of unique pairwise interactions occurring in each dataset is also indicated.

At the regional level, we found a total of 345 unique combinations of plant–pollinator interactions considering both visitation and pollen-transport networks, from which 127 (36.8%) were found in both types (figure 2, electronic supplementary material, table S5). From a total of 222 unique pairwise interactions detected in the pollen-transport metanetwork, 95 (42.8%) were exclusive to it (i.e. they were not registered in the visitation metanetwork, electronic supplementary material, table S6) and 123 out of 250 (49.2%) were recorded only in the visitation metanetwork (electronic supplementary material, table S7). Furthermore, we identified important differences in the number of interactions established by some plant species in both metanetworks (electronic supplementary material, tables S8 and S9). The most outstanding case was *K. arvensis,* which was visited by 19 different bees but only four of them (all bumblebees) transported its pollen.

Additionally, we found that the presence of an interaction in both metanetworks (i.e. visitation and pollen transport) was affected by the plant habitat specialization and the pollinator group. Specifically, interactions involving habitat specialist plants (*χ* ^2^ = 6.47, d.f. = 1, *p* = 0.011) and bumblebees (*χ* ^2^ = 17.24, d.f. = 1, *p* = 0.0071) had a higher occurrence in both metanetworks than those involving habitat generalist plants and solitary bees (electronic supplementary material, figure S3). Flower size, bee size and bee habitat specialization had no significant effects on the presence of an interaction in both metanetworks (electronic supplementary material, table S10).

**Figure 3.**
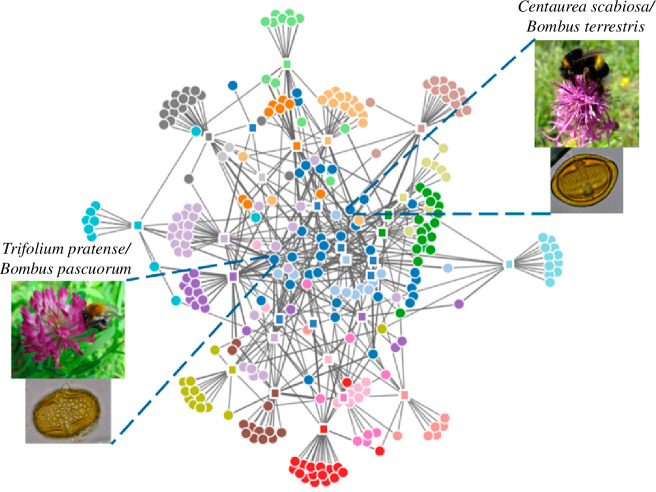
Pollen-transport metanetwork structure among calcareous grassland fragments and unique pairwise pollen–bee interactions (*n* = 29 and *n* = 222, respectively). Circles indicate pairwise pollen–bee interactions and squares represent grassland fragments. Interactions occurring in at least two fragments form links between sites. Thickness of links (grey lines) is proportional to interaction abundance. Colours represent metanetwork modules based on the Walktrap community-finding algorithm (igraph package) [66]. This algorithm indicates the presence of subgraphs that constitute a distinctive community. Nodes with greater centrality occur in the central positions of the graph [67].

In the visitation metanetwork, we found 76 (30.4%) unique pairwise interactions that occurred in at least two calcareous grassland fragments but these accounted for 913 (78.4%) interaction events. In the pollen transport metanetwork (figure 3), we found 70 (31.5%) unique pairwise interactions occurring in at least two fragments but summing up to 452 (72.2%) interaction events. Finally, we found a significant positive effect of landscape diversity on the number of single-fragment interactions for both network types (electronic supplementary material, figure S4). However, the spatial scale at which this effect was stronger differed for the visitation and pollen-transport networks. Specifically, the number of single-fragment interactions increased with landscape diversity at the 150 m scale for the visitation data (*χ* ^2^ = 4.59, d.f. = 1, *p* = 0.032, electronic supplementary material, figure S4*a*) and at the 500 m scale for the pollen transport data (*χ* ^2^ = 5.96, d.f. = 1, *p* = 0.015, electronic supplementary material, figure S4*b*). Moreover, landscape diversity at the 500 m scale significantly increased the proportion of single-fragment interactions (*F*_1,27_ = 5.26, *p* = 0.030), but this effect was only found for the pollen-transport networks (electronic supplementary material, figure S5). Fragment area and fragment connectivity had no significant effect on the number of single-fragment interactions or the proportion of single-fragment interactions (electronic supplementary material, table S11).

## 4. Discussion

In this study, we analysed multiple levels of ecological complexity of plant–pollinator networks constructed from bee–flower visitation and bee–pollen-transport interactions across a gradient of habitat fragmentation of a biodiversity hotspot. Of all interactions found, 63.2% were exclusive to either the visitation or pollen-transport networks, highlighting both the numerous low-frequency interactions that are not captured by observations of flower visits (27.5%) and the high number of interactions (35.7%) that do not translate into pollen transport. Pollen-transport networks were more specialized than visitation networks, and a higher diversity of land cover types in the surroundings of a habitat fragment decreased network specialization.

### (a) Network type effects on network specialization

Ecological and methodological aspects can affect the relationship between the specialization of pollen-transport and visitation networks. Regarding ecology, an important aspect is whether studies consider all flower-visiting taxa [31–33,68] or rather focus on bees [29,49]. Different from other flower-visiting arthropods, female bees have specialized structures for pollen transport (i.e. scopae) and actively search for pollen to feed their larvae. As bees carry more pollen than other flower visitors [25,69], the chance of detecting rare interactions with pollen analyses is higher for bees. These bee traits would favour the higher presence of transport-only interactions and the lower presence of visitation-only interactions compared to other pollinator taxa. On the other hand, as central-place foragers, bees are bound to the areas surrounding their nest location, and they therefore can have a lower probability of carrying pollen from plants not belonging to the local habitat community compared to other flower visitors, such as butterflies that can move more freely through the landscape. This implies that the pollen that bees carry has a higher chance of being a subset of the flowers observed in a focal study site than is the case in other taxa. This difference is expected to be especially important in bee communities with many small- and medium-sized solitary bees, but even larger bees like *Apis* spp. and *Bombus* spp. are known to forage mostly in proximity to their nests when enough flowering resources are available, which is usually the case in calcareous grasslands [70–72]. In line with our findings, previous bee studies using microscopy have found higher specialization of pollen-transport networks compared to visitation networks [29], or no significant difference between them [49], suggesting a prevalence of the later phenomenon compared to the former.

Regarding methodology, several aspects can affect specialization indices. First, if plant species recorded through pollen but missing in the study sites are not excluded from the analyses, there would be an overrepresentation of interactions in the pollen-transport networks and an underrepresentation of detected interactions in the visitation networks. This would increase the probability of finding pollen-transport networks to be more generalized than visitation ones. In addition, the lower resolution of plant taxa identification by light microscopy or metabarcoding, compared to field observations of flower visits, needs to be taken into account. This can be done, for example, by lowering the resolution of the plant visitation data from species to genus level for those species that cannot be discriminated based on pollen morphology or DNA analyses.

The location on the bee's body from which pollen is removed to construct pollen-transport networks can also affect the results. Previous studies disagree on removing pollen located in the corbiculae from the family Apidae [29,31,33,68], pollen located in the scopae [23,25], or considering all carried pollen [32,49]. The exclusion of pollen located in specialized bee structures can considerably reduce the number of detected interactions. Further, most studies based on microscopy report network specialization to be higher in pollen-transport networks compared to visitation networks [23,25,29,68], while studies based on metabarcoding report the opposite [32,33,73]. A possible reason for this effect is that metabarcoding results can vary depending on the plant reference database used, eventually inflating false detections [73]. Light microscopy also has limitations, including the expertise of the observer and the possibility of lower taxonomic resolution than metabarcoding [74]. Lastly, the use (or not) of a threshold to distinguish pollen contamination from legitimate pollen transport [25,33,49] also affects the number of interactions found and can therefore impact specialization estimations. The methodological approach used in our study was designed to minimize pitfalls related to spurious interactions detection and to maximize the reliability of results.

A higher specialization of pollen-transport networks in calcareous grasslands indicates that these pollination networks might be more vulnerable to collapse following disturbance because increased specialization can make pollination networks less robust and more prone to co-extinction cascades [75]. The vast majority of plant–pollinator network studies are based on visitation data and conclusions regarding biodiversity conservation are derived mostly from them. In light of our results and previous studies [23,25,29,68], we call for attention to the risk of an overestimation of plant–pollinator networks' stability and robustness in past studies based solely on flower visitation data. Still, whether the higher specialization of pollen transport with respect to visitation networks is the rule across different habitats and geographical locations and how much is it affected by methodological artefacts remain to be investigated.

### (b) Landscape diversity effects on network specialization

Plant–pollinator network specialization is affected by species richness and species behaviour [35,75,76]. Habitat fragmentation and landscape simplification may decrease the availability of interacting partners as a consequence of reduced population sizes or local extinctions [77]. The absence of interacting partners can have opposite effects on species specialization. On the one hand, pollinators may visit more plant species to compensate for missing resources, therefore increasing their generalization [78]. However, in case of low behavioural plasticity or high plant fidelity, specialization could increase after disturbance (i.e. due to the loss of a plant partner) as pollinators would be unable to establish new interactions. For plants, losing a pollinator may directly increase plant specialization by reducing the number of interacting partners. Nonetheless, reduced competition for resources among pollinators could facilitate visitation of opportunistic (and usually less effective) pollinators, therefore increasing plant generalization [78].

In protected natural and seminatural habitats, mutualistic networks present higher nestedness and specialization than expected by chance, reducing interspecific competition through niche partitioning and allowing coexistence [8,79,80]. Habitat fragmentation and extreme climatic events reduce mutualistic network specialization through the loss of specialized and rare interactions [8,76], thereby increasing the relative proportion of interactions involving generalist species [6]. Jauker *et al*. [35] also reported this pattern when analysing plant–pollinator networks in calcareous grasslands. They found that habitat loss decreased network specialization through the loss of species and interactions, resulting in small and tightly connected networks. Given the pronounced process of fragmentation of European calcareous grasslands in the past century [38,81], it is expected that the remaining, commonly small and isolated fragments have already lost many specialized and rare interactions, as shown for butterflies on the same calcareous grasslands [82].

Habitat generalist bees can use floral and nesting resources of several land cover types and may establish opportunistic interactions with multiple (habitat generalist and specialist) plant partners. In our study, landscape diversification stands for high variety of land cover types in the matrix, dominated by oilseed rape, grainfields, maize, pastures, grasslands, field margin strips and forest stands. Any increase in landscape diversification in the surrounding of a calcareous grassland habitat fragment (e.g. matrix diversification) may increase the influx (i.e. spillover) of habitat generalist species establishing new generalized interactions, further decreasing grassland network specialization. Hence, specialization would be reduced by increasing the number of potential interaction partners available, closing the gap between the potential and the realized number of interacting partners of each species.

Accordingly, habitat loss and landscape simplification may have opposite effects on European calcareous grasslands network specialization by affecting groups of species with different traits. Habitat loss may cause a selective reduction in richness or abundance of species with low behavioural plasticity and high plant fidelity (i.e. specialists), resulting in small and homogeneous networks with an overrepresentation of generalist species. In contrast, landscape simplification may result in a reduction of species with high behavioural plasticity and low plant fidelity (i.e. generalists), resulting in fragile and specialized networks. The most plausible mechanism that could explain the patterns found in this study is that pollinators did not compensate for the missing plant partners and that plants did not get extra visits once a pollinator was lost following landscape homogenization, resulting in more specialized interaction networks.

### (c) Visitation and pollen-transport unique interactions

The large proportion of flower visitor species transporting few or no pollen, due to morphological or behavioural constraints, is an intrinsic characteristic of pollination systems [23,25,29,49]. However, it is important to note that this proportion is usually reported to be larger in light microscopy studies than metabarcoding studies [74]. The selection of a threshold of pollen grains carried by a bee as proof of legitimate pollen transport (in opposition to accidental transport due to contamination) is fundamental to avoid false positives, however, it is also non-trivial [22]. Popic *et al*. [29] used a 10 grains threshold and reported only 38% of visitation links resulting in pollen transport (51% in our study). Manincor *et al*. [49], also studying bee interactions in calcareous grasslands of Europe (threshold five pollen grains), reported doubling the total number of interactions found (40% increase in our study) when looking at pollen transport (i.e. pollen-transport only interactions). Our approach was conservative; the choice of a lower threshold would have most likely augmented the differences found. Despite the more conservative threshold used in our study, the structural differences found between bee visitation and pollen-transport networks are in accordance with the literature and challenge the assumption that visitation data are sufficient surrogates of animal-mediated pollen transport [29,49].

The large presence of flower visitors with a relatively small capacity for pollen transport raises many questions regarding their importance for pollination [83]. In theory, deposition of a single conspecific pollen grain could be enough for pollination to occur, but pollen deposition thresholds are common given that not all pollen deposited by pollinators is viable [84]. Therefore, a relatively high amount of conspecific pollen deposition is usually needed for a meaningful pollination success [84]. The concomitant deposition of heterospecific pollen is also an important factor considering its negative effects on pollination [85,86]. Actually, from a plant species perspective, a strategy based on maximizing pollinators’ visits might come at the cost of high heterospecific pollen deposition on their stigmas. Contrastingly, a strategy based on the attraction of a small number of specialized pollinators (and therefore larger potential for conspecific pollen deposition) comes at the cost of a higher dependence on a small group of pollinators, with a higher risk of local extinction and a lower probability of visitation. Habitat fragmentation and landscape homogenization may impose a reduced set of pollinator partners to interact with. Consequently, higher plant specialization could arise as an indirect result of the lack of alternative partners and not as part of an ecological strategy to increase reproductive success.

The pollen-transport networks revealed a high amount of rare interactions. This implies that plant–pollinator networks based only on flower visitation data are not just biased by the inclusion of interactions with no potential for pollination, but also by missing many rare interactions. Consequently, pollen loads analysis represents an important complementary approach to study pollination systems because the actual pollen dispersal across the plant community can be quantified. Visitation data, on the other hand, appear fundamental to understand plant–pollinator interactions from the pollinator perspective, as competition among pollinators and the different foraging strategies that pollinators use to maximize their fitness can be analysed. For example, bees visit some flowers exclusively to collect nectar and eventually may not enter into contact with flower anthers. These interactions may not be important from the plant perspective because pollen transport may not occur, but they are still fundamental for bees as they will influence their diet. The detection of interactions involving rare habitat specialist plants, such as *Scabiosa columbaria* [87,88], indicates that pollen load analyses can contribute to improve conservation strategies by identifying remaining small populations of these rare species. For example, restoration efforts targeting these small populations could be undertaken in places where the plants were thought to be locally extinct.

### (d) Plant habitat specialization and pollinator group

Bumblebees in central Europe have a high capacity for pollen transport given that they are abundant, larger than most solitary bees and have dense hair [36,89,90]. Our results support this by demonstrating that the probability of a bumblebee carrying pollen after a flower visit is higher than that of solitary bees. However, studies on pollen transfer (i.e. pollen deposition on a conspecific stigma) after flower visits would be necessary to test whether bumblebees are also able to deposit more pollen on stigmas than solitary bees, since pollen transport does not always translate into pollen deposition [19]. For example, the pollen located in specialized structures, such as the corbiculae, is generally assumed not to be available for pollination [91]. This may not make a difference in pollen transport analyses [49], but it could be important for pollen transfer to stigmas. Although bumblebees are hairier than other bee groups, which is associated with a higher pollination effectiveness [36], plants' pollination also depends on pollinator behaviour, flower morphology and on the ratio between conspecific and heterospecific pollen deposition [85,92]. Therefore, solitary bees may be irreplaceable for the reproductive success of some plant species.

In fact, solitary bees were found to be fundamental for the pollen transport of many plant species. In particular, they transported pollen from 16 habitat specialist plants and were the only pollen vector for three of them. Considering that many solitary bee species are vulnerable and threatened with extinction [53,93], these results signal to the importance of their role in calcareous grasslands and to the potential risk of their absence for habitat specialist plants' reproductive success. Our findings reveal that both bumblebees and solitary bees are complementary for pollen transport of calcareous grasslands plant species.

We found a smaller representation of habitat generalist than habitat specialist plants on pollen-transport networks in calcareous grasslands. Hence, flower visits to habitat specialist plants have a higher probability to translate into pollen transport than visits to habitat generalist plants. The habitat generalist *K. arvensis*, for example, was visited by many species of both bee guilds (bumblebees and solitary bees), being involved in a total of 105 interaction events. However, we found only 10 interactions with *K. arvensis* in the pollen-transport networks, involving only four bumblebee species and no solitary bees. In contrast, pollen from the habitat specialist *Onobrychis viciifolia* was transported by all of its seven species of visitors from both bee guilds.

A higher representation of habitat specialist plants in pollen-transport networks cannot be solely related to a higher attractiveness of habitat specialist flowers or pollen, as interactions involving habitat specialist plants in the pollen-transport dataset were less than half of the total interactions found (46.8%). This result is rather a consequence of different mechanisms that allow habitat specialist plants to allocate their pollen more frequently on flower visitors than habitat generalists. Habitat specialist plants are expected to have a long history of evolutionary adaptations to the local pollinator pool and, therefore, to have developed mechanisms for efficient pollen transport through those pollinators [37]. Conversely, generalist plants should lack such adaptations, as they would exhibit more opportunistic strategies to quickly adapt to different environments. The adaptations of plants to increase pollination success can occur at many levels, including pollen vector attraction, pollen presentation, pollen transport and pollen germination [94]. At the visitation level, traits such as flower size, flower abundance and the quantity and quality of offered flower rewards (i.e. pollen and nectar) may increase visitation rates [95]. At the pollen transport level, plants may possess mechanisms to place larger amounts of pollen at specific places on the flower visitors’ body [94]. At the pollen transfer level, plant traits such as the stigma type (i.e. wet or dry), pollen morphological traits or behavioural characteristics of pollinators may affect the quantity and quality of pollen deposition [19,94,96]. Even after pollen deposition on stigmas, plants may exhibit mechanisms to regulate receptiveness depending on the characteristics of the flower visitor [97].

### (e) Landscape diversity effect on single-fragment interactions

The spatial scale at which landscape diversity most strongly affected the number of single-fragment interactions was larger for the pollen-transport networks compared to the visitation networks. This suggests that landscape-scale conservation measures to protect plant–pollinator networks might be undertaken at the wrong spatial scales when solely based on flower visitation data. The increased number of single-fragment interactions with landscape diversity is not solely related to a general increase in the total number of interactions with landscape diversification but rather that landscape diversification has a disproportionately positive effect on the occurrence of single-fragment interactions compared to the total amount of interactions. Importantly, this effect was only captured with the pollen transport data and highlights that landscape structure effects can remain undetected in plant–pollinator studies solely based on visitation data. In contrast to a previous study in the area [11], the effect of landscape diversity on the proportion of single-fragment interactions could be explained by the central-place foraging behaviour of bees. Differently from mobile butterflies, bees may determine that the positive effects of landscape diversification at small spatial scales do not spread through the metanetwork but rather increase the local diversity of plant–pollinator interactions in single fragments.

## 5. Conclusion

By analysing plant–pollinator networks across a gradient of habitat fragmentation, we found that pollen-transport networks were more specialized than visitation networks, indicating that plant–pollinator networks could be more vulnerable than previously believed. Only 36.8% of the total number of registered plant–pollinator interactions occurred in both flower visitation and pollen-transport networks. Our landscape analysis of a pollen-transport metanetwork also revealed that the properties of pollination networks are affected by landscape diversity at scales that differ from those informed by visitation networks, which may increase the accuracy and effectiveness of landscape-level measures for the conservation of plant–pollinator networks. Interactions involving habitat specialist plants and bumblebees had a higher probability of simultaneously occurring in the visitation and pollen-transport networks than interactions involving habitat generalist plants and solitary bees. Nonetheless, the pollen of several plant species was found to be transported only by solitary bees. Our study shows that conservation of pollination systems and related pollination services needs finer data on the biological processes underlying plant–pollinator interaction networks, such as pollen load analyses. Our metanetwork approach allowed us to identify rarity of plant–pollinator interactions and local uniqueness, which can be further used by local authorities to design tailored conservation strategies. Our results have important consequences for the understanding of the responses of plant–pollinator networks to habitat fragmentation and contribute to unveiling important processes underpinning the dynamics of these networks.

## Data Availability

All data associated to this manuscript can be accessed at [98]. Electronic supplementary material is available online [99].
